# Hands-on immunology: Engaging learners of all ages through tactile teaching tools

**DOI:** 10.3389/fmicb.2022.966282

**Published:** 2022-08-25

**Authors:** Felix R. Harris, Michael L. Sikes, Michael Bergman, Carlos C. Goller, Andrew O. Hasley, Caroline A. Sjogren, Melissa V. Ramirez, Claire L. Gordy

**Affiliations:** ^1^Department of Biological Sciences, North Carolina State University, Raleigh, NC, United States; ^3^Biotechnology Program, North Carolina State University, Raleigh, NC, United States; ^2^Department of Chemical and Biomolecular Engineering, North Carolina State University, Raleigh, NC, United States

**Keywords:** tactile teaching tool-guided inquiry learning, antibody, epitope, MHC, haplotype, universal design for learning, inclusive teaching, active learning

## Abstract

Ensuring the public has a fundamental understanding of human–microbe interactions, immune responses, and vaccines is a critical challenge in the midst of a pandemic. These topics are commonly taught in undergraduate- and graduate-level microbiology and immunology courses; however, creating engaging methods of teaching these complex concepts to students of all ages is necessary to keep younger students interested when science seems hard. Building on the Tactile Teaching Tools with Guided Inquiry Learning (TTT-GIL) method we used to create an interactive *lac* operon molecular puzzle, we report here two TTT-GIL activities designed to engage diverse learners from middle schoolers to masters students in exploring molecular interactions within the immune system. By pairing physical models with structured activities built on the constructivist framework of Process-Oriented Guided Inquiry Learning (POGIL), TTT-GIL activities guide learners through their interaction with the model, using the Learning Cycle to facilitate construction of new concepts. Moreover, TTT-GIL activities are designed utilizing Universal Design for Learning (UDL) principles to include all learners through multiple means of engagement, representation, and action. The TTT-GIL activities reported here include a web-enhanced activity designed to teach concepts related to antibody–epitope binding and specificity to deaf and hard-of-hearing middle and high school students in a remote setting and a team-based activity that simulates the evolution of the Major Histocompatibility Complex (MHC) haplotype of a population exposed to pathogens. These activities incorporate TTT-GIL to engage learners in the exploration of fundamental immunology concepts and can be adapted for use with learners of different levels and educational backgrounds.

## Introduction

Our previous work demonstrated that Tactile Teaching Tools with Guided Inquiry Learning (TTT-GIL) can provide an effective approach to teaching complex topics in microbial molecular genetics in diverse student populations. Introduction of an interactive, 3D *lac* operon puzzle resulted in significant learning gains in both populations in which it was implemented, with a larger effect size in a genetics class at an underfunded minority-serving institution than in a microbiology class at an R1 primarily white institution (Gordy et al., 2020; Ramirez and Gordy, 2020). The inclusivity of TTT-GIL activities, which are created using Universal Design for Learning (UDL), along with their hands-on, game-like nature, suggest that they may be an effective way of teaching cellular and molecular immunology concepts even to younger students (Hall et al., 2003; Rose and Meyer, 2006; Burgstahler and Cory, 2010; Rappolt-Schlichtmann et al., 2012; Meyer et al., 2014; Ramirez and Gordy, 2020). We therefore developed and implemented two additional TTT-GIL activities focused on microbiology and immunology concepts: one targeted to middle and high school students, and one targeted to advanced undergraduates and masters students.

The first TTT-GIL activity presented focuses on interactions between viral epitopes and antibodies. This activity is modified from a published resource designed for in-person undergraduate courses (Suchman et al., 2018). The activity presented here has been adapted for use with middle and high school students at the North Carolina School for the Deaf (NCSD) in Morganton, North Carolina. After aligning the learning outcomes (LOs) with the NC Standard Course of Study (NC Standard Course of Study, 2022a, b,c), the lesson was modified to allow for implementation *via* Zoom, with digital materials provided *via* a website (Goller, 2022),^1^ and physical models mailed to the students. While the activity was created for students working with American Sign Language (ASL) interpreters in a synchronous online setting, we believe it is appropriate for use with any middle or high school population and can be effectively adapted for use in-person or in a blended setting.

The second TTT-GIL activity presented focuses on interactions between Major Histocompatibility Complex (MHC) molecules and peptide antigens, how MHC haplotypes impact population survival in the presence of pathogens, and how pathogens in turn shape the population’s MHC haplotype over evolutionary time. This novel activity was developed for a 400-level Immunology course at NC State University, and could be adapted for use in microbiology, evolution, or population genetics courses. Pre- and post-assessment results demonstrate learning gains for concepts aligned with the LOs defined for the activity, and also suggest future modifications to deconstruct misconceptions.

## Antibody activity

### Activity goals and summary

To engage middle and high school students in learning about antibody-epitope interactions, we modified a published activity developed for use in undergraduate microbiology courses (8, Supplementary Table S1). In the adapted activity, we chose to focus on two of the LOs included with the original activity:

1. *Describe* how antigens and epitopes are related using examples of virus antigens.
2. *Explain* why some antibodies that do not bind to epitopes are produced.

These LOs align with the NCSCS for Middle School and High School Science (NC Standard Course of Study, 2022b,c) as described in the instructor notes.

The activity was carried out during a virtual outreach event in which students, teachers, and ASL interpreters at NCSD interacted with NC State instructors *via* Zoom. Models and handouts were mailed to the students prior to the event, and students were asked to watch videos describing fundamental concepts addressed in the activity on the event website^2^ and complete a pre-activity worksheet^3^ ahead of time.

During the event, instructors guided a discussion, answered questions about the pre-activity worksheet, and then asked students to work in groups to further explore their models through the TTT-GIL activity. The activity ended with questions and answers between the students and instructors.

Full instructions for printing and assembling activity kits, along with instructor notes, slide sets and student handouts, are available at https://stembuild.ncsu.edu/lesson-plan/antibodies-epitopes .

### Antibody and virus models

The influenza and antibody heavy and light chain (HC and LC) models described by Suchman et al. and the SARS-CoV2 viral particle published by Alexander Thomas were printed using a LulzBot Mini 2 3D printer (Suchman, 2014; Thomas, 2020). The viral particles were printed using 2.85-mm polylactic acid (PLA) filament, while the HC and LC models were printed with flexible thermoplastic urethane (TPU) filament to allow the antibody molecules to slightly bend, more accurately representing the antibody-epitope interaction.

Antibodies were assembled by gluing magnets to the circular indentations of each HC and LC and attaching the LCs to the HCs. The HCs and LCs were numbered so that they could be reassembled if needed. Students were provided with assembled antibodies with a small, conical binding pocket (HC1 + LC1) or a larger, spherical binding pocket (HC2 + LC2).

**Figure 1:**
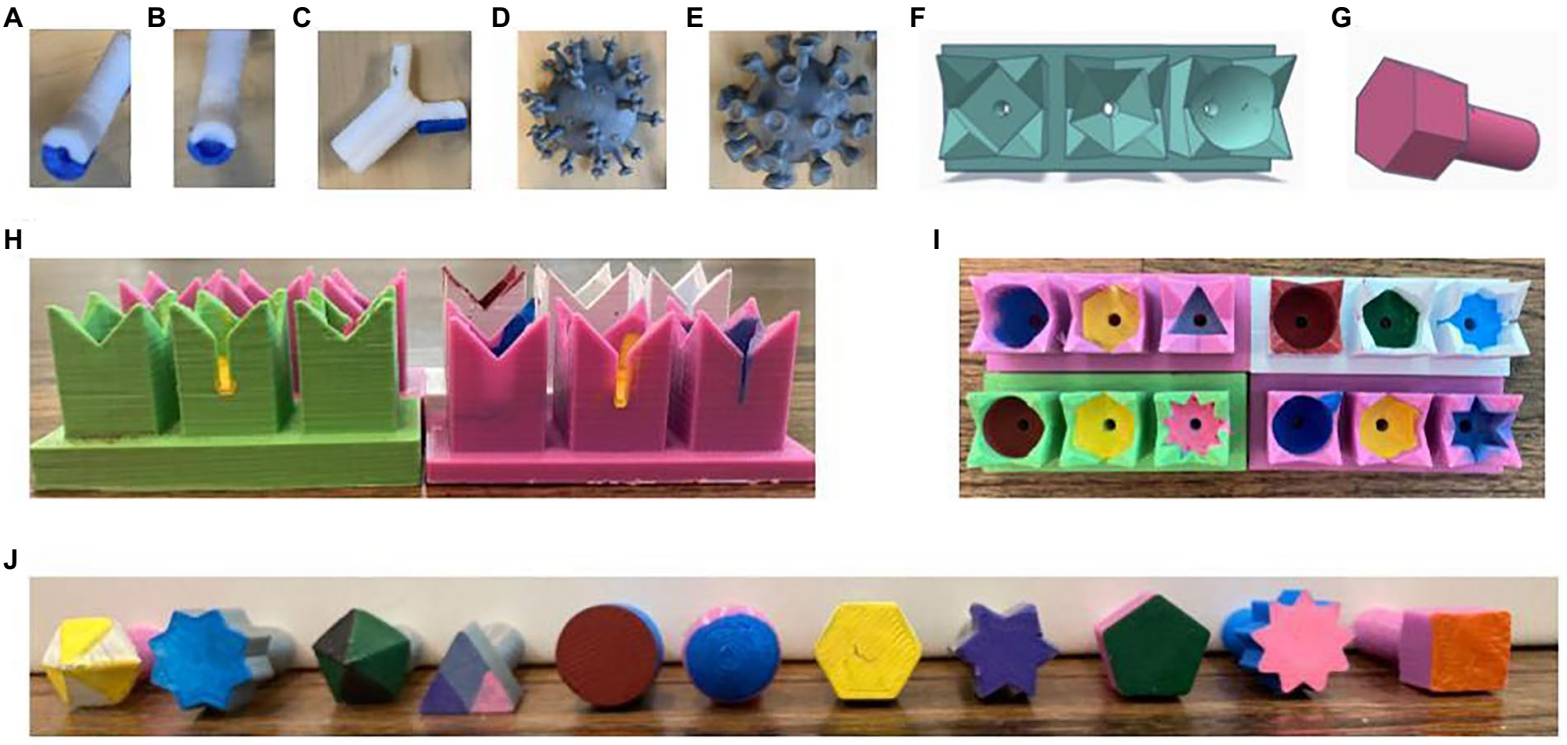
Tactile teaching tools. **(A**,**B)** Assembled thermoplastic urethane (TPU) antibodies with conical/small binding pockets (**A**, Antibody 1) or spherical/large binding pockets (**B**, Antibody 2). **(C)** Antibody with one detachable light chain removed. **(D)** Influenza viral particle. **(E)** SARS-CoV2 viral particle. **(F)** Tinkercad rendering of an MHC array. **(G)** Tinkercad rendering of a peptide antigen. **(H,I)** Front **(H)** and top **(I)** views of four different MHC arrays. **(J)** Front view of all 11 peptide antigens.

Activity kits containing one influenza, one Antibody 1, and one Antibody 2 per group as well as one SARS-CoV2 per class were mailed to the students prior to the activity (Figure 1).

### Activity

#### Pre-activity videos and worksheet (Part 1)

Prior to the event, students were asked to watch two videos (2–3 min each) posted on a website created for the outreach event (Goller, 2022).^4^ The first video was created in Canva using 3D renderings of pathogens and antibodies purchased from Getty Images or downloaded from the Protein Data Bank (PDB). The goal of this video was to introduce students to the shapes and relative sizes of viral particles and antibodies, and to provide examples of antibodies binding to viral epitopes, resulting in viral neutralization. The second video was a publicly available video created by Rachel Taylor that explains the function of the immune system in fighting pathogens and defines key terms, including pathogens, antigens, antibodies (Taylor, 2020).^5^ The information in these videos is further explained in a text- and image-based format on the event website^6^ to provide multiple means of representation and engagement (Hall et al., 2003; Rose and Meyer, 2006; Burgstahler and Cory, 2010; Rappolt-Schlichtmann et al., 2012; Meyer et al., 2014).

Students were then asked to complete parts 1a and 1b of the handout prior to the event.

#### Guided inquiry learning activity (Part 2)

The activity was carried out *via* Zoom and structured with a set of Google Slides^7^ to guide students as they interacted with the 3D models. Throughout the activity, care was taken to provide sufficient time for ASL interpreters to relay information to students and for students to raise questions *via* the Zoom chat or the interpreters. The activity began with a review of antibody structure, followed by discussion of the students’ answers to pre-activity questions.

The questions posed as the students began to work with the models differed from those used in the published resource. As a GIL activity, the students began by identifying the parts of the model (how many different viral spike types can you find? which part of the antibody is the HC? which is the LC?). The students were then asked to determine which viral epitope antibody 1 (conical/small binding pocket) and antibody 2 (spherical/large binding pocket) could bind. After they were given time to test the binding of their antibody to various epitopes, a 30-s pre-recorded idealized demonstration of testing binding using the models was shown.

The students were next asked to create new antibodies by combining LC1 with HC2 and vice versa, and to determine how this affected the binding ability of their antibodies. Finally, the students were asked to attempt interactions between each of their original antibodies and the SARS-CoV2 model.

These activities led into a series of discussion questions aimed at generalizing the concepts addressed by the activity (available in Instructor Notes). Notably, the activity allowed multiple means of expression, as students could communicate *via* Zoom chat, ASL interpreters, or by using their models to ask or answer questions, and they were able to express their understanding through writing on the pre-activity worksheet (Hall et al., 2003; Rose and Meyer, 2006; Burgstahler and Cory, 2010; Rappolt-Schlichtmann et al., 2012; Meyer et al., 2014).

### Classroom management and modifications

The activity is designed to be completed in 30–45 min. The instructor may choose to show the pre-activity videos and ask students to work alone or in groups on the pre-activity worksheet in class if the class period is sufficiently long. For students not working with ASL interpreters or for more advanced students, the activity may be completed faster.

Instructors may wish to provide additional pathogens for students to interact with. Viral and bacterial models can be found on Thingiverse (2022) as well as the NIH 3D Print Exchange (2022). Instructors should note that models shared on these websites are created at different scales, and scales should be manually adjusted prior to printing.

When discussing the pre-activity videos, instructors may wish to remind students that the interactions between cells of the immune system and pathogens are described as a “war” in order to reference something that they know about and understand, but in fact, these are chemical interactions.

Suggestions for assessment include reflections, concept quizzes, and questions that ask students to label heavy and light chains, constant and variable regions, antigen binding sites, and epitopes.

## Major histocompatibility complex activity

### Activity goals and description

This activity was designed for a 400-level Immunology course with the following specific LOs:

1. *Differentiate* between individual and community haplotypes
2. *Predict* how MHC haplotype will be shaped over evolutionary time by a pathogen

The activity was carried out during an optional review session on the evolution of MHC haplotypes within a population. Students worked in groups of four, with each group receiving four 3D-printed arrays of 3 MHC molecules and a bag of 3D-printed antigens. The groups completed a series of GIL activities focused on three goals. First, they determined the prevalence of different MHC molecules within their group and within the class. Second, they determined which antigens were able to interact with which MHCs. Third, they determined how the diversity of the class-wide MHC haplotype changed following an outbreak of a pathogen that “killed” any individuals whose MHC molecules were unable to bind pathogen-derived antigens.

### Major histocompatibility complex and antigen models

Eleven different antigen models, each represented by a different geometric shape, and arrays of three attached MHC models, each with a binding pocket represented by a geometric shape corresponding to the shapes used for the antigen molecules, were designed in Tinkercad Dashboard (2022). Classroom sets were printed with LulzBot Mini 2 3D printers using 2.85-mm PLA filament. The antigens and binding pockets were further differentiated by painting the binding surfaces different colors (Figure 1).

A total of 24 different types of arrays were created, each with a unique combination of three different binding pockets. The classroom set of MHC arrays was designed such that some binding pockets were common among the population, and others were rare, representing the relative abundance of different MHC molecules within the MHC haplotype.

Additionally, the MHC and antigen molecules were designed such that some antigen molecules were able to interact with more than one type of MHC binding pocket – for example, the pentagon antigen will fit within the pentagon binding pocket or the hexagon binding pocket, but the hexagon antigen will only fit within the hexagon binding pocket. STL files and kit assembly instructions are available at https://stembuild.ncsu.edu/resource/MHC-haplotype, and instructor notes, handouts, and group haplotype sheets are available at https://stembuild.ncsu.edu/lesson-plan/MHC-haplotype.

### Major histocompatibility complex haplotype evolution activity

#### Identification of MHC molecules

Students were asked to form groups of four and select a reporter. Each group received a bag including four MHC arrays, 11 peptide antigens, a set of numbered index cards, one activity handout per student, and a single group haplotype sheet used to record the MHCs present within the group. Each group member identified and circled the shape and color of the binding pocket of each of the three MHC molecules within their array. After comparing individual haplotypes among the group, the reporter tallied the total numbers of each MHC binding pocket present within their group in the first table of the group haplotype sheet (Supplementary Table S2).

The instructor gathered this data from each group to calculate the abundance of each of the MHC binding pockets within the entire class and facilitated a discussion of the most and least common MHCs. Based on the number of different MHC binding pockets present, the students were then asked to make a prediction about the number of different antigens that could be presented by the MHC molecules in the population. Generally, students predicted that the number of antigens that could be presented was equal to the number of different MHC molecules in the population.

#### Testing antigen binding

Each group member was next asked to test the ability of each of the 11 peptide antigens to bind to each of the 3 MHC molecules within their array, and to mark any possible interactions (antigens able to fit in the binding pocket) in table 2 of their handout (Supplementary Table S3). Each group shared their findings internally and then with the class. This discussion led students to conclude that the number of peptide antigens that could be presented was greater than the number of MHC molecules present in the population because some of the MHC binding pockets were able to accommodate more than one antigen. The instructor then asked the students whether they predicted the frequencies of MHC alleles within the population were fixed, or whether they would change over time.

#### Outbreaks

In the next parts of the activity, students were instructed that a series of outbreaks occurred. In the first outbreak, a completely unvaccinated population with no access to antivirals and antibiotics was exposed to smallpox (the multicolored triangle peptide antigen). The students were asked to determine whether they were able to mount an adaptive immune response to the pathogen, and whether they lived or died. Each group’s reporter then completed table 2 on the group haplotype sheet to again tally the total numbers of each MHC binding pocket present within their group, but this time only recording the MHC molecules present in surviving individuals.

The instructor then collected this information from the groups, and the class discussed how the class wide MHC haplotype had or had not changed before moving on to the next outbreak. In the second outbreak, the population was exposed to Ebola, which was processed into two different peptides for presentation by MHC-I: the pink 9-pointed star and the green and black pentagon. Survivors of the smallpox outbreak were asked to again determine whether they were able to mount an immune response and survive. This allowed for a discussion of which individuals would survive: only those whose MHC alleles allowed them to present both antigens, or also those who were able to present at least one antigen. The reporter collected data from the group, and the composition of the classwide MHC haplotype was updated.

Finally, the population was exposed to a *Mycobacterium tuberculosis* outbreak. The few surviving students determined whether they survived *M. tuberculosis*, the reporter collected their data, and the class discussed the final outcome of this series of outbreaks.

### Classroom management

This activity was carried out in one 75-min class period. It could be modified to be carried out in a 50-min class period by completing any lecture or instruction needed to provide background knowledge during a prior class session and/or dividing the activities into multiple class sessions. For example, students could identify their MHC haplotypes and test their ability to recognize the 11 peptide antigens in one class period, and simulate outbreaks in a second class period. The role of the instructor is to facilitate group discussion and sharing of data collected within the groups. In the class in which we implemented this activity, reporters held up an index card numbered 0–4 to indicate the number of individuals in the group with a given MHC molecule in their array.

Questions were posed using the TopHat polling system (for example “Are you alive or dead?”), and results were provided to the class as graphs in real time, and then discussed. Depending on the size of the class and technology available, other methods of collecting data and managing classroom discussion may be more appropriate.

### Evidence of learning and modifications

To determine whether the activity impacted students’ learning of concepts aligned with the LOs, consenting participants were asked to complete an anonymous pre-assessment prior to the in-class activity. The same questions were asked again on their exam, and learning gains were measured by comparing the percent of students answering the question correctly on the pre-assessment to the percent of students answering the same question correctly on the post-assessment (Supplementary Document 1).

Of the four aligned assessment questions, students showed gains of more than half of a standard deviation (Cohen’s d ≥ 0.5) for two, questions 2 and 4 **(**Supplementary Data File**)**. Notably, students demonstrated an effect size of 0.50 for question 4 despite a high starting level of understanding from which to improve, with 85.6% of the students answering this question correctly on the pre-assessment (Table 1). This finding demonstrates a strong ability to predict how MHC haplotype is shaped over time by pathogens (LO 3). Fewer students answered question 2 correctly on the pre-assessment (61.5%); however, a learning gain of approximately two-thirds of a standard deviation (Cohen’s d = 0.64) resulted in 92% of students answering the question correctly on the post-assessment (Table 1), indicating increased ability to differentiate between individual and community-level MHC haplotypes (LO 1).

**Table 1:** Learning gains in students completing the MHC haplotype activity.

	LO	Pre-assessment, % correct	Post-assessment, % correct	Corrected effect size (Cohen’s *d*)
2. Although there are hundreds of MHC alleles in the human population, each person inherits only a small number of MHC alleles.	1	61.5%	92%	0.64
4. Infections with a high mortality can change a population’s MHC allele frequency in subsequent generations.	2	85.6%	100%	0.50
6. The specific MHC molecules expressed by an individual will not change over time as that individual is exposed to pathogens.	1, 2	76.9%	69%	−0.14
7. The specific MHC molecules expressed by a population will change over time as that population is exposed to pathogens.	1, 2	92.3%	92%	0

The percent of students answering each of four assessment questions correctly before (pre) and after (post) the activity. The pre-assessment was carried out in class immediately before the activity, and the post-assessment was included on an exam in the course. Effect size was measured using Cohen’s *d* with a correction for sample size, and was calculated as follows: [(Mean Post–Mean Pre)/Pooled SD] × [(*N* − 3)/(*N*–2.25)] × [(*N*−2)/*N*]1/2, where pooled SD = [(SDPre^2^ + SDPost^2^)/2]^½^, *N*, number of people in that group (Durlak, 2009).

The remaining two assessment questions may reveal a misconception that could be addressed in future iterations of this activity. While 92% of students correctly stated on both the pre- and post- assessments that the MHC molecules expressed by a population will change over time as the population is exposed to pathogen exposure, fewer students correctly stated that the MHC molecules expressed by an individual will *not* change over time (Table 1). Based on this finding, we suggest that instructors modify the handout to add a question to each of the outbreak sections asking students not just to determine whether their MHC molecules are able to bind the pathogen-derived peptide antigen and whether they will live or die, but also whether the composition of their individual MHC haplotype will change.

## Summary

These activities provide examples for instructors who wish to engage students of all ages and levels in constructivist learning of complex concepts related to microbiology and immunology. Moreover, we demonstrate a novel strategy that leverages an activity website and TTTs to facilitate a remote active learning session with physical models. Together with Shoaf et al.’s findings shared in this issue demonstrating the effectiveness of a TTT-GIL activity implemented in a hyflex environment (Shoaf et al., under review)^8^, our experience provides further evidence that hands-on microbiology and immunology activities can be used to reach learners outside of a traditional classroom setting.

## Author’s note

For the printing of the models, the safety guidelines of the specific 3D printer being used should be followed. Possible risks associated with 3D printing include heat, electrical, mechanical, and fume risks. There are no risks associated with the use of the assembled models in the classroom setting. This study was performed following a protocol that was approved by the NC State University (NCSU) Institutional Review Board for the Use of Human Subjects in Research (protocol number 12731).

## Data availability statement

The datasets presented in this study can be found in online repositories. The names of the repository/repositories and accession number(s) can be found at: STEM BUILD Database: https://stembuild.ncsu.edu/lesson-plan/antibodies-epitopes; https://stembuild.ncsu.edu/lesson-plan/MHC-haplotype; and https://stembuild.ncsu.edu/resource/MHC-haplotype.

## Ethics statement

The studies involving human participants were reviewed and approved by North Carolina State University Institutional Review Board. The patients/participants provided their written informed consent to participate in this study.

## Author contributions

FH designed, printed, and painted the MHC and peptide models, assembled activity kits, contributed to activity design, facilitated the classroom activity, contributed to data analysis, and edited the manuscript. MS developed the idea for the MHC activity, contributed to activity design, facilitated the classroom activity, designed the assessment questions, contributed to data analysis, and edited the manuscript. MB, CCG, AH, and CS adapted the published antibody activity, aligned the activity to NCSOS, created the event website and all materials, assembled antibody activity kits, facilitated the antibody activity, and edited the manuscript. MR contributed to development of the MHC activity, aligned the MHC activity, learning outcomes, and assessments, facilitated the classroom activity, designed the assessment questions, contributed to data analysis, and edited the manuscript. CLG contributed to development of the MHC activity, aligned the MHC activity, learning outcomes, and assessments, facilitated the classroom activity, designed the assessment questions, contributed to data analysis, advised MB, CCG, AH, and CS on development of the antibody activity, assembled flexible heavy and light chain models with magnets, and wrote the manuscript. All authors contributed to the article and approved the submitted version.

## Funding

Development and implementation of the MHC activity was supported by an internal grant to MR and CLG from North Carolina State University Digital Education and Learning Technology Applications. FH was supported by an NC State Provost’s Professional Experience Program fellowship. Development and implementation of the antibody activity was supported by NSF award 2018668 RCN-UBE INCUBATOR STEM BUILD: A network of undergraduates, faculty, and makers utilizing 3D printing to build understanding through inclusive learning design to MR and CLG. CCG, AH, and CS were supported by an NIH Innovative Program to Enhance Research Training (IPERT) grant “Molecular Biotechnology Laboratory Education Modules (MBLEMs)” 1R25GM130528-01A1.

## Conflict of interest

The authors declare that the research was conducted in the absence of any commercial or financial relationships that could be construed as a potential conflict of interest.

## Publisher’s note

All claims expressed in this article are solely those of the authors and do not necessarily represent those of their affiliated organizations, or those of the publisher, the editors and the reviewers. Any product that may be evaluated in this article, or claim that may be made by its manufacturer, is not guaranteed or endorsed by the publisher.

## Footnotes

^1^ https://go.ncsu.edu/antibody

^2^ https://sites.google.com/ncsu.edu/antibody/intro

^3^ https://sites.google.com/ncsu.edu/antibody/handout

^4^ https://go.ncsu.edu/antibody

^5^ https://youtu.be/qCRwuxDpthY

^6^ https://sites.google.com/ncsu.edu/antibody/part-1

^7^ https://sites.google.com/ncsu.edu/antibody/part-2

^8^Shoaf, P. T., French, K. S., Clifford, N. J., McKenney, E. A., Ott, L. E. (under review) A gut microbiome guided-inquiry and tactile tool activity promotes student learning. *Front. Microbiol*.
