# Supplementary material for: Hands-on immunology: Engaging learners of all ages through tactile teaching tools
Source: Front Microbiol. 2022 Aug 25;13:966282. doi: 10.3389/fmicb.2022.966282 (PMC9453673; doi:10.3389/fmicb.2022.966282)
Supplement: Supplementary file 1 [file Table_1.DOCX]

#

|  | **Suchman *et al.* activity** | **Modified antibody activity** |
| --- | --- | --- |
| Target group | Undergraduates | Middle-high school students |
| Learning Outcomes | 1. Describe how antigens and epitopes are related. 2. Explain why some antibodies that do not bind to epitopes are produced. 3. Discuss which regions on the heavy and light chains come together to bind to specific epitopes. 4. Identify the region on the antibody that determines its class or isotype. | 1. Describe how antigens and epitopes are related using examples of virus antigens. 2. Explain why some antibodies that do not bind to epitopes are produced. |
| Mode of instruction | In-person | Online, synchronous (Zoom) |
| Classroom characteristics | Flipped classroom | Deaf/Hard of Hearing students supported by ASL interpreters |
| 3D models | Influenza virus (PLA)  Conical heavy chain/HC 1(PLA)  Conical light chain/LC 1 (PLA)  Spherical heavy chain/HC 2 (PLA)  Spherical light chain/LC 2 (PLA) | SARS-CoV2 virus (PLA)  Influenza virus (PLA)  Conical heavy chain/HC 1 (TPU)  Conical light chain/LC 1 (TPU)  Spherical heavy chain/HC 2 (TPU)  Spherical light chain/LC 2 (TPU) |
| Activity summary | Multi-part activity presented as a challenge for learners to explore antigen-antibody interactions. | Web-enhanced learning experience that leverages TTT and website as framework for dissecting the antigen-antibody model. Focus on accessibility and guided inquiry. |

**Table S1.** Comparison of the resource published by Suchman et al. and the modified antibody activity described here.

#

| **MHC #1:** | **MHC #2:** | **MHC #3:** |
| --- | --- | --- |
| Red Circle:  Blue Sphere: | Yellow Hexagon:  Green Pentagon:  White 3D Hexagon:  Black 3D Pentagon: | Periwinkle Triangle:  Purple 6-pointed star:  Blue 8-pointed star:  Pink 9-pointed star:  Orange Square: |

**Table S2.** MHC recording table (Table 1) from Group Haplotype Sheet. For each of the three positions within each array, the group’s recorder tallied the number of group members with each type of binding pocket at that position. Tables 2, 3, and 4 from the Group Haplotype Sheet are identical to this table, except that they contain an additional column asking the reporter to record the number of individuals in their group who survived the outbreak. The full Group Haplotype Sheet is available at <https://stembuild.ncsu.edu/lesson-plan/MHC-haplotype>.

|  | **MHC #1 shape:** | **MHC #2 shape:** | **MHC #3 shape:** |
| --- | --- | --- | --- |
| Red circle |  |  |  |
| Green and black  3D pentagon |  |  |  |
| Blue 8-pointed star |  |  |  |
| Multicolored triangle |  |  |  |
| Orange square |  |  |  |
| Purple 6-pointed star |  |  |  |
| Yellow and white 3D hexagon |  |  |  |
| Yellow hexagon |  |  |  |
| Pink 9-pointed star |  |  |  |
| Green pentagon |  |  |  |
| Blue sphere |  |  |  |

**Table S3.** Antigen binding table (Table 2) from individual MHC activity handout. Each student first recorded the shape of each of their three MHC molecules’ binding pockets, and used check marks to indicate which peptides were able to bind each of the three MHC molecules.
