# Supplementary material for: Hands-on immunology: Engaging learners of all ages through tactile teaching tools
Source: Front Microbiol. 2022 Aug 25;13:966282. doi: 10.3389/fmicb.2022.966282 (PMC9453673; doi:10.3389/fmicb.2022.966282)

The pre-assessment below was given to students immediately before beginning the activity. The same questions were included on their exam and served as a post-assessment. While all questions aligned with the learning outcomes of the course and of the optional review session in which the MHC haplotype TTT-GIL activity was implemented, questions 2, 4, 6, and 7 aligned with the learning objectives specific to the TTT-GIL activity and were used to evaluate the effectiveness of the activity.

**MHC Activity Pre-Assessment**

**NAME ________________________________________________**

**This assessment is not for a grade.**

1. What is the function of MHC I and MHC II molecules?
   1. They provide cytokine stimulation to activate αβ T lymphocytes
   2. They present peptide antigens to αβ T lymphocytes
   3. They provide “signal 2” to activate αβ T lymphocytes
   4. They help phagocytose bacterial pathogens
2. Which statement is true about the genes that encode human MHC proteins?
   1. The ability to activate a wide variety of T lymphocytes is generated by V(D)J recombination of the MHC genes.
   2. The ability to activate a wide variety of T lymphocytes is encoded in the hundreds of different MHC alleles that each person inherits.
   3. Although there are hundreds of MHC alleles in the human population, each person inherits only a small number of MHC alleles.
   4. The human MHC genes (or HLA genes) are edited by AID during the processes of positive and negative thymocyte selection.
3. Which statement about MHCs is true?
   1. MHC I, expressed by most cells in the body, binds cytoplasmic peptide antigens for activation of CD8 T cells, while MHC II, primarily expressed only by professional antigen presenting cells, binds peptide antigens from membrane or extracellular sources for activation of CD4 T cells.
   2. MHC I, primarily expressed only by professional antigen presenting cells, binds peptide antigens from membrane or extracellular sources for activation of CD4 T cells, while MHC II, expressed by most cells in the body, binds cytoplasmic peptide antigens for activation of CD8 T cells.
4. How does the distribution of MHC alleles vary with the spread of pathogens?
   1. Infections with a low mortality will select the loss of a particular MHC allele from a population’s gene pool.
   2. Infections with a high mortality are most likely to involve antigens that bind multiple MHCs.
   3. Infections with a high mortality can change a population’s MHC allele frequency in subsequent generations.
   4. MHC alleles are generated by V(D)J recombination and are not influenced by infections.
5. MHC II is formed by assembly of one alpha and one beta chain, each of which help form the shape of the antigen-binding pocket. In contrast, the antigen-binding pocket of MHC I is exclusively formed by the MHC I heavy chain. What does this mean about MHC I genetics?
   1. Antigen specificity of each MHC I is encoded by two genes.
   2. Each MHC I allele binds only one peptide antigen.
   3. MHC I alleles are inherited, which MHC II alleles are assembled by V(D)J recombination.
   4. A person heterozygous for MHC I (ie, they inherited two different alleles of HLA-A, HLA-B and HLA-C), would express a total of only 6 different MHC-I antigen-binding shapes.
6. True or false: The specific MHC molecules expressed by an individual will change over time as that individual is exposed to pathogens.
   1. True
   2. False
7. True or false: The specific MHC molecules expressed by a population will change over time as that population is exposed to pathogens.
   1. True
   2. False
8. To the best of your ability, define “MHC haplotype”.
9. The graphs below represent the prevalence of different HLA-A alleles in a human population at two different time points, before and after the population experienced an epidemic of an infectious disease that had not been encountered before. Based on the data in these graphs, select the answer that provides the most likely explanation for the differences in these graphs.
   1. HLA-A*02:02 and HLA-A*02:04 molecules were not able to bind peptides derived from the pathogen, and individuals expressing these MHCs were more likely to die.
   2. HLA-A*02:02 and HLA-A*02:04 molecules were able to bind peptides derived from the pathogen, and individuals expressing these MHCs were more likely to die.
   3. HLA-A*02:189 molecules were not able to bind peptides derived from the pathogen, and individuals expressing this MHC were more likely to survive.
   4. HLA-A*02:189 molecules were able to bind peptides derived from the pathogen, and individuals expressing this MHC were more likely to survive and reproduce.

Before: After:


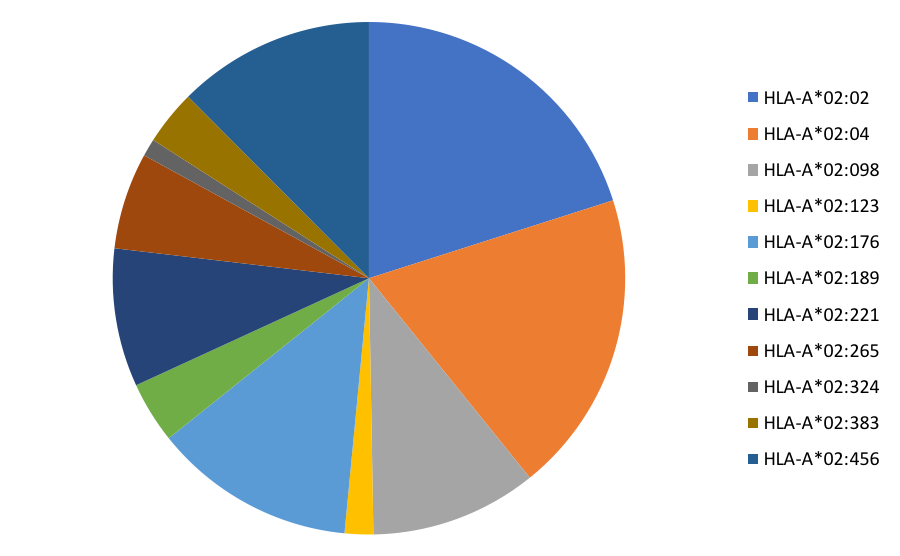

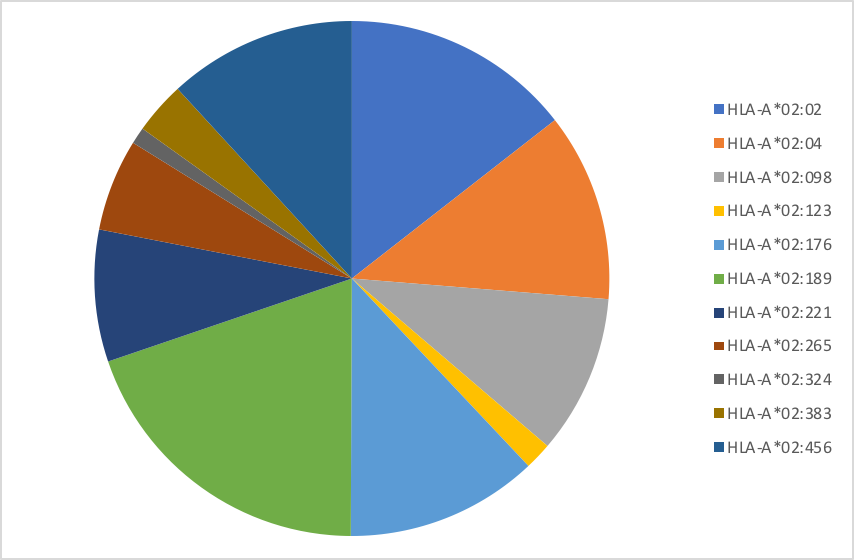

Supplement: Supplementary file 2 [file Table_2.DOCX]
